# Biomarkers in Vestibular Schwannoma–Associated Hearing Loss

**DOI:** 10.3389/fneur.2019.00978

**Published:** 2019-09-18

**Authors:** Luis Lassaletta, Miryam Calvino, Jose Manuel Morales-Puebla, Pablo Lapunzina, Lourdes Rodriguez-de la Rosa, Isabel Varela-Nieto, Victor Martinez-Glez

**Affiliations:** ^1^Department of Otorhinolaryngology, La Paz University Hospital, Madrid, Spain; ^2^IdiPAZ Research Institute, Madrid, Spain; ^3^Centre for Biomedical Network Research on Rare Diseases (CIBERER), CIBER, Institute of Health Carlos III, Madrid, Spain; ^4^Institute of Medical and Molecular Genetics (INGEMM), La Paz University Hospital, Madrid, Spain; ^5^Institute for Biomedical Research “Alberto Sols” (IIBM), Spanish National Research Council-Autonomous University of Madrid (CSIC-UAM), Madrid, Spain

**Keywords:** vestibular schwannoma, neurofibromatosis type 2, biomarkers, hearing loss, perilymph, chemokine, heat shock protein, genotype

## Abstract

Vestibular schwannomas (VSs) are benign tumors composed of differentiated neoplastic Schwann cells. They can be classified into two groups: sporadic VS and those associated with neurofibromatosis type 2 (NF2). VSs usually grow slowly, initially causing unilateral sensorineural hearing loss (HL) and tinnitus. These tumors cause HL both due to compression of the auditory nerve or the labyrinthine artery and due to the secretion of different substances potentially toxic to the inner ear or the cochlear nerve. As more and more patients are diagnosed and need to be managed, we are more than ever in need of searching for biomarkers associated with these tumors. Owing to an unknown toxic substance generated by the tumor, HL in VS may be linked to a high protein amount of perilymph. Previous studies have identified perilymph proteins correlated with tumor-associated HL, including μ-Crystallin (CRYM), low density lipoprotein receptor-related protein 2 (LRP2), immunoglobulin (Ig) γ-4 chain C region, Ig κ-chain C region, complement C3, and immunoglobulin heavy constant γ 3. Besides, the presence of specific subtypes of heat shock protein 70 has been suggested to be associated with preservation of residual hearing. It has been recently demonstrated that chemokine receptor-4 (CXCR4) is overexpressed in sporadic VS as well as in NF2 tumors and that hearing disability and CXCR4 expression may be correlated. Further, the genetic profile of VS and its relationship with poor hearing has also been studied, including DNA methylation, deregulated genes, growth factors, and *NF2* gene mutations. The knowledge of biomarkers associated with VS would be of significant value to maximize outcomes of hearing preservation in these patients.

## Introduction

Vestibular schwannomas (VSs), previously termed acoustic neuromas, are non-malignant tumors composed of Schwann cells of the vestibulocochlear nerve (VIII cranial nerve), arising from either the internal auditory canal (IAC) or the cerebellopontine angle (CPA). They can be classified into two groups: sporadic VS and those associated with neurofibromatosis type 2 (NF2) (1). Most VSs are sporadic (90%), with a combined lifetime risk of 1:1,000 for developing a unilateral tumor (1, 2). Nowadays, a higher rate of VS diagnosis has been described, due to incidental findings from magnetic resonance imaging (MRI) performed due to unrelated complaints (3).

The mortality rate of VS ranges from 0.2 to 1% (4). Due to their anatomic position, patients suffer from progressive hearing loss (90%) and tinnitus (>60%), with facial numbness (12%) and facial paralysis (6%) occurring mainly among patients with larger lesions. Balance dysfunction is often present, although <20% of patients manifest with vertigo symptoms (1, 2). VSs usually grow slowly, leading to unilateral sensorineural hearing loss (SNHL) and tinnitus, theoretically caused by compression of the auditory nerve (3) or by spasm or occlusion of the labyrinthine artery (5). Sudden SNHL may also be the clinical presentation in up to 22% of the cases (6). These tumors may cause hearing loss also due to the secretion of substances potentially toxic to the inner ear or the cochlear nerve (1, 7, 8).

Diagnosis of VS may be either from cranial MRI performed for unrelated complaints or, usually, due to unilateral hearing loss or tinnitus (1, 9).

Audiological tests including audiometry and auditory brainstem response are not reliable predictors of CPA pathology (10). As most patients with CPA tumors have a comparable set of symptoms and audiometric results, the diagnosis relies mainly on imaging. The gold standard for the diagnosis of VS is MRI with gadolinium (1). In the last years, improvements in technology and a higher accessibility to MRI have increased the number of diagnosed VSs (11).

Approximately three of each of the four VSs exhibit no growth, leading to an observation strategy (wait and scan). Mean growth is about 2–4 mm/year in growing tumors (12). The hearing status, the growth rate, the subject's age, and the surgeon's experience are the main factors when deciding between surgery (destructive or conservative) and gamma knife therapy (13). In recent years, gamma knife radiosurgery is becoming a more popular choice for subjects with growing small or medium tumors and useful hearing, while patients with large-size tumors usually undergo surgery (14).

As more and more patients are diagnosed and need to be managed, we are more than ever in need of searching for biomarkers associated with these tumors, in order to help with the choice of selecting between a “wait-and-scan” approach and surgery (15) aimed at reducing morbidity and increasing the hearing outcomes (4).

## Biological Markers

Due to the lack of biopsy sampling without the destruction of the organ, little is known about the cellular and molecular correlates in inner ear pathology (16).

In recent years, considerable progress in proteomics has been enabled by modern technology. By a shotgun proteomics approach, the identification of proteins with high sensitivity is enabled (17). Mass spectrometric (MS) analyses have been successfully used for auditory proteomics and require a more concerted effort for biomarker identification (18). In addition, sophisticated methods for perilymph sampling (19) have revolutionized the field of otology, offering precious biofluid samples for analysis.

### Perilymph Proteome

Perilymph, an extracellular fluid of the inner ear, is found within the scala tympani and vestibuli of the cochlea. During an apoptotic or necrotic episode inside the inner ear, the proteins that are secreted can be found at high concentrations in this fluid (20). The knowledge of the perilymph proteome may shed some light on the mechanisms of tumor-associated hearing loss, which are mostly unknown to date (15). Stankovic et al. speculated that because of an unknown toxic substance generated by the VS, hearing loss in VS could be linked to a high protein concentration in the perilymph (7).

#### Perilymph Proteins Related to Hearing Loss

In 2011, Lysaght et al. identified 15 proteins from perilymph specimens (selected by comparing VS and cochlear implant samples) with differential expression and biological function. They suggested the use of this list in future research focused on distinguishing between better vs. worse hearing in patients with VS (see Table 1) (20).

**Table 1:** Proposed biomarkers of human VSs related to worse hearing.

***CRYM***	μ-Crystallin
*FN1*	Fibronectin 1
*KRT10*	Keratin 10
APOC3	Apolipoprotein C-III
VCAN	Versican
DCD	Dermcidin
SERPINB12	Serpin family B member 12
CTSD	Cathepsin D
SERPINB3	Serpin family B member 3
SERPINA5	Serpin family A member 5
SOD3	Superoxide dismutase 3, extracellular
PARK7	Parkinson disease protein 7
SERPINF1	Serpin family F member 1
CHI3L1	Cartilage glycoprotein 39
**LRP2**	Low-density lipoprotein receptor-related protein 2

*Modified from Lysaght et al. (20)*.

μ-Crystallin (CRYM) or nicotinamide adenine dinucleotide phosphate (NADPH)-regulated thyroid hormone binding protein is located within the cytoplasm, where it promotes transcription of the thyroid hormone triiodothyronine (T3) (20, 21). *CRYM* gene mutations cause autosomal-dominant hearing loss due to changes in the intracellular localization and the inability to bind to T3, which may lead to an altered K+ recycling (20, 22).

Low density lipoprotein receptor-related protein 2 (LRP2) or megalin is a trans-membrane receptor protein, which can be found in certain epithelial cells such as those of the ear. LRP2 has the ability to bind several ligands, being essential in the process of endocytosis of different elements such as sterols, lipoproteins, hormones, and vitamin binding proteins. Two well-known conditions, Donnai-Barrow and facio-oculo-acoustico-renal (FOAR) syndromes (23), both associated with SNHL, are the result of mutations in the *LRP2* gene (20).

On the other hand, of the 91 commonly identified perilymph proteins of patients with VS on an individual level, Rasmussen et al. described four proteins that were significantly associated with tumor-related deafness: Immunoglobulin (Ig) γ-4 chain C region, Ig κ chain C region, complement C3, and immunoglobulin heavy constant γ 3. These 91 proteins were identified in 12 out of 15 samples they used in the study (15), which was confirmed by analogy with data from previous MS research on perilymph (20, 24).

Moreover, alpha-2-HS-glycoprotein, a suggested inflammatory and immunological intermediary in perilymph, was suggested to be associated with deafness in patients with SVs. It was also discovered in samples from VS patients in 2017 (24), and although its concentration was not directly linked to the hearing outcomes, the authors attempted to further investigate this potential association. Rasmussen et al. hypothesized that VS may excrete alpha-2-HS-glycoprotein to the perilymph, where its inflammatory activity may lead to SNHL. Factors elicited from the VS may also affect the inner ear, inducing an upregulation of alpha-2-HS-glycoprotein within the perilymph (15).

#### Heat Shock Proteins and Hearing Loss

Heat shock proteins (HSPs) are stress proteins, which mediate cell survival under critical environmental conditions (25). Increased perilymph levels of 10 different subgroups of HSP were detected in subjects undergoing cochlear implantation that preserved hearing when compared with those without hearing preservation, and cochlear transcriptome data suggest that there is a baseline protective expression of HSP70 1A, 1B, 2, 4, 5, 6, 8, 9, and 12A mRNA (16).

HSP90 is the most important chaperone for cellular stress. It is involved in pathological processes, such as cancer development (26), and its increased expression as a stress responsive biomarker is present in multiple types of tissue inflammation (27).

Recently, Schmitt et al. found that HSP90 was determined in the perilymph of half of the patients (*n* = 18) experiencing complete loss of residual hearing loss after cochlear implantation, whereas only one of the patients with preserved residual hearing showed HSP90 in perilymph. The upregulation of HSP90 in the perilymph may therefore induce the migration of macrophages and leukocytes, resulting in cochlear inflammation. However, despite the cellular changes observed, the authors could not detect a significant difference in HSP90 expression in patients with VS compared with patients without tumor (16).

On the other hand, HSP70 has been identified as an otoprotective agent and protects hair cells from stress-induced apoptosis (28). Interestingly, the presence of some subtypes of HSP70 seemed to correlate with preservation of residual hearing in cochlear implantation (16). It has been associated with an increase in the cell proliferation rate (29) and, according to Schmitt et al., could take part in the development of VS, despite the authors not finding any correlation between HSP70 expression and VS when comparing with subjects without tumor (16). One explanation could be the low proliferation rate of these tumors; by contrast, medulloblastomas, fast-proliferating intracranial tumors with poor prognosis, showed an increased expression of HSP70 (29).

According to these findings, more data on the regulation of these proteins and perilymph proteomics are mandatory to demonstrate the role of these HSPs in patients with VS and hearing loss.

#### Increased Concentration of Perilymphatic Proteins and MRI Findings

Increased signal intensity of the fluid on three-dimensional fluid-attenuated inversion recovery (3D-FLAIR) MRI has been reported in various diseases, including SNHL, and VS (30–33). An increased concentration of proteins in the perilymphatic space has been proposed to explain the enhanced cochlear signal on FLAIR images in subjects with VS (34–37).

Kim et al. demonstrated a correlation between a higher cochlear signal on 3D FLAIR images and hearing loss in patients with VS (38). The correlation was stronger in intrameatal tumors when compared to all subjects, and no correlation was found when considering only CPA tumors. Interestingly, the cochlear signal intensity on MRI was significantly higher in tumors confined to the IAC.

#### Endolymphatic Hydrops

Ipsilateral inner ear alterations, including endolymphatic hydrops (EH) and acidophilic-staining precipitate, have been observed in temporal bone histopathological studies from patients with VS (5, 39). In the past years, intratympanic gadolinium injection has arisen as a new tool in the diagnosis of EH (40). Recently, delayed intravenous gadolinium-enhanced high-resolution MRI of the inner ear has been shown to provide resolution adequate for accurate detection of EH (41, 42). In addition, heavily weighted T2 sequences are useful to evaluate the cochlear fluids in patients with VS. In patients with a tumor entirely blocking the IAC, the volume of the vestibular endolymphatic space can be determined with great certainty. Venkatasamy et al. described a difference in the perilymphatic signal on a T2-weighted steady state free precession acquisition at 3T, providing a new tool for differentiating schwannomas and meningiomas (43). In patients with VS, a correlation between the endolymphatic space volume and the level of hearing loss has been described. Eliezer et al., using a 3D non-contrast T2 heavily weighted sequence at 3T, showed that the utricle volume was correlated with the patient's hearing loss in a series of 23 VSs. As most subjects with VS benefit from a wait-and-scan strategy, based on these MRI results, they suggest that a treatment leading to decreased EH could be administrated to achieve better hearing outcomes (44). In a recent paper (45), a saccular dilatation on the ipsilateral side was demonstrated in 30% of VSs (53 out of 183 patients with typical VS). In this study, a 3D non-contrast high-resolution T2-weighted sequence was used.

### Tissue Sample Proteins

#### Cytokines and Hearing Loss

A large number of cytokines are produced by tumors (46) including VSs. To maintain the homeostasis in the cochlea, the cytokine balance is of vital importance (47). Like other substances, cytokines have been suggested to play a role in the labyrinth of degenerative changes (5). However, few studies have examined the role of these proteins in VS (48, 49).

Chemokine receptor-4 (CXCR4) is implicated in several pathological processes, including autoimmune disease, infection, and tumor development (50, 51). CXCR4 is overexpressed in many neoplasms, being capable of increasing tumor growth and invasiveness (52, 53).

C-X-C Motif Chemokine Ligand 12 (CXCL12), a ligand for CXCR4, cooperates with metastatic cells to CXCL12-expressing organs. The Ras/Raf/MEK and the PI3K/Akt/mTOR pathways can be activated by CXCL12 binding to the CXCR4 receptor. In a similar way, the loss of Merlin (52, 53), a tumor suppressor protein encoded by the *NF2* gene in VS (54), leads to activation of these two primary pathways.

Recently, Breun et al. have described that CXCR4 could play a role in the pathogenesis of both sporadic and NF2-associated VS. In their study CXCR4 was overexpressed in these tumors, with no significant differences found between the two groups. CXCR4 mRNA expression increased with the degree of hearing loss when compared with the control group, with the results lacking statistical difference (53).

Although tumor extension may be related to hearing impairment in VS (55), there is usually a discrepancy between tumor size and hearing disability (9). A reason why hearing disability is not always correlated with tumor dimensions could be an invasive growth pattern caused by CXCR4 overexpression in certain tumors. Indeed, Breun et al. detected no correlation between CXCR4 expression and tumor extension; therefore, this chemokine receptor may be significant for tumor invasiveness, as exhibited by hearing disability (53).

### Molecular Biology

According to Celis-Aguilar et al.'s review (8), the molecular biology of VS could been explained by several pathogenic mechanisms including chromosome 22 loss, *NF2* gene mitotic recombination (56), DNA methylation (57), deregulation of genes (58), immunogenic factors (59), cytokines, and growth factors (60–62), and *NF2* gene mutation (63).

#### DNA Methylation

Epigenetic alterations are found across many solid cancers, and although most efforts in VS are limited to the controversial DNA methylation of the *NF2* gene, other changes have shown to play an important role in VS. Lassaletta et al. investigated the methylation status of 16 genes in 22 sporadic VSs and related it to clinical and radiological findings (57), the connection observed between *TP73* aberrant methylation and deafness being important [Pure Tone Average (PTA) = 43 and 17 dB for patients with methylated and unmethylated *TP73* genes, respectively]. A genome-wide methylation analysis in VS also showed a trend toward hypomethylation in several miRNAs and coding genes, including alternative transcripts, opening a window to possible therapeutic targets (64).

#### Deregulated Genes

In a study searching for associations between the molecular basis of VS and hearing loss (7), surgical specimens of these tumors from 13 patients were classified into two groups based on gene expression, one with good hearing (word recognition >70% and PTA ≤ 30 dB) and another with poor hearing. *PEX5L, RAD54B*, the prostate-specific membrane antigen-like gene, and *PSMAL* had low expression in VS patients with bad hearing outcomes. Besides, the *CEA-CAM7* gene and Carcinoembryonic Antigen (CEA) protein were overexpressed in VS patients with poor hearing (8).

#### Growth Factors

The development of VS has also been associated with abnormal expression of growth factors. In a study of tumor samples from 11 subjects with VS, Lassaletta et al. described an inverse correlation between the expression of platelet-derived growth factor A and deafness (65). On the other hand, vascular endothelial growth factors (VEGFs) have been associated with the hearing status of patients with VSs (66, 67). Most VSs express VEGF, and it has been suggested that this growth factor may play a role in both tumor growth and hearing status (68, 69). Bevacizumab, a VEGF neutralizing antibody, was used by Plotkin et al. to treat patients with VS, an increase in hearing reported in four out of seven subjects treated with this drug (67). In recent years, bevacizumab has been reported to increase speech understanding and hearing quality in several NF2 patients (68, 70).

#### NF2 Gene Mutation

*NF2* gene mutations have been associated with the hearing level of patients with VS. In the study of Lassaletta et al., 51 cases undergoing surgery for VS were analyzed. Patients with *NF2* gene mutations presented lower PTA thresholds compared with nonmutated cases (71).

Selvanathan et a1. analyzed the impact of age of onset on the existence of several NF2-related symptoms, including hearing impairment or tinnitus, and found that there was a significantly younger age of onset of symptoms in patients with nonsense or frameshift mutation (i.e., mutations that produce protein truncation). They hypothesized that a younger age of onset of VS could explain the younger age of onset of hearing loss (and tinnitus) (72).

Halliday et al. proposed a genetic severity score (1, tissue mosaic; 2A, mild classic; 2B, moderate classic; and 3, severe) in order to predict morbidity for NF2 subjects in certain dimensions including hearing status (73). According to Emmanouil et al., if subjects were stratified according to genetic severity, it could help to obtain a better prognostication of the hearing decline. In their study, they described a significant difference in terms of hearing decline according to the genetic severity: the median age for subjects rated as “severe” was 32 years, compared to a median of 80 years for patients classified as “tissue mosaic” (74).

## Conclusion

So far, no reliable methods are able to predict the evolution of hearing loss in subjects with VS. Several markers such as perilymph proteins have been associated with tumor hearing loss. Also, specific subtypes of HSP70 have been correlated with hearing outcomes. Cytokines produced by VS, especially CXCR4 expression, have been related to hearing impairment. DNA methylation, deregulated genes, growth factors, and *NF2* gene mutation have also been related to hearing loss in subjects with VS. Most of these potential markers of hearing loss are not routinely available for the clinician. On the other hand, recent findings on imaging, especially delayed intravenous gadolinium-enhanced high-resolution MRI, and 3D non-contrast heavily T2-weighted sequences are promising in terms of therapeutic management of patients with VS showing signs of EH. The precise knowledge of biomarkers associated with hearing loss in patients with VS would be useful to minimize morbidity and to maximize outcomes of hearing in these patients.

## Author Contributions

All authors listed have made a substantial, direct and intellectual contribution to the work, and approved it for publication.

### Conflict of Interest Statement

The authors declare that the research was conducted in the absence of any commercial or financial relationships that could be construed as a potential conflict of interest.
